# Infra-low frequency neurofeedback in persistent postural-perceptual dizziness—Case report

**DOI:** 10.3389/fnhum.2022.959579

**Published:** 2022-07-22

**Authors:** Roxana Sasu

**Affiliations:** Neurotopia, Woodland Hills, CA, United States

**Keywords:** persistent postural-perceptual dizziness, infra-low frequency, neurofeedback, vertigo, psychosomatic disorder, balance

## Abstract

Persistent Postural-Perceptual Dizziness, also known as PPPD or 3PD, is a chronic functional vestibular disorder characterized by persistent sensation of rocking or swaying unsteadiness and/ or non-spinning dizziness without vertigo lasting at least 3 months. Symptoms typically worsen with upright posture (like standing or sitting upright), head or body motion and exposure to busy or visually rich environments. The article describes the application of Infra-Low Frequency Neurofeedback (ILF NF) over 32 sessions on an unmedicated individual with symptoms related to PPPD that were still present 3 years after the initial diagnosis. Along with significant reduction in those symptoms, other accompanying symptoms (like anxiety, intrusive violent thoughts, suicidal thoughts) were markedly improved with ILF NFB. Consistent symptom tracking from session to session, as well as before and after CPT data were used to document reported changes with ILF NF.

## Neurofeedback as an alternative intervention

Infra-Low Frequency Neurofeedback, a non-invasive brain exercise technique meant to promote self-regulation and optimal brain function, has been used on a 27-year-old male, to impact a variety of symptoms that were part of his complex clinical presentation at the beginning of training in 2021. Some of these symptoms had a sudden onset or worsening in early 2019, leaving this client struggling with debilitating anxiety, brain fog, constant rocking or bobbing sensation, among other things, impairing his day-to-day functioning in every aspect of his life. Medical consults and testing didn't reveal any structural problems, and SSRIs were prescribed as the best course of treatment at the time. The client preferred an alternative route to treatment and started vestibular rehabilitation in 2020, which provided considerable relief from some of his symptoms. The audiologist used the term Persistent Postural-Perceptual Dizziness to refer to his set of symptoms. While there was no ICD-10 (10^th^ edition of the International Statistical Classification of Disease and related Health Problems) code for Persistent Postural-Perceptual Dizziness, two codes could be combined to best categorize the symptoms: F45.9 (psychosomatic disorder) ICD 10 (2021a) and R42 (vertigo) ICD 10 (2021b). In 2017 PPPD was defined by the International Society for Neuro-otology, the largest international society on dizziness (Staab et al., 2017), and that same year it was added to the 11^th^ edition of the International Statistical Classification of Diseases and related Health Problems (ICD-11) (WHO, 2015), (Bisdorff et al., 2015). PPPD has been extensively researched in recent years, but epidemiologic data is not easily obtainable, given the fact that the disorder was newly redefined, and patients will see a variety of specialty physicians before being diagnosed with it. A UK study of primary care found that 4% of all patients registered with a general practitioner experience persistent symptom of dizziness, most being incapacitated by their symptoms (Nazareth et al., 1999).

PPPD is classified as a chronic functional vestibular disorder (Staab et al., 2017). It is not a psychiatric or structural condition. It is characterized by persistent sensation of rocking or swaying unsteadiness and/ or dizziness without vertigo lasting at least 3 months. Interestingly, these symptoms often occur in a nervous system already suffering with anxiety, depression or somatic symptoms, and these comorbidities tend to worsen the dizziness temporarily (Staab et al., 2017).

Adding Neurofeedback to his treatment regimen was recommended by the audiologist working with him, to address the anxiety and depression-related symptoms the patient was still struggling with. These were not impacted by the vestibular rehabilitation exercises he had completed in the program. The expectation was that Neurofeedback would have a positive impact on anxiety and depression-related symptoms, as well as the remaining sensory processing and integration concerns related to his diagnosis. The client had been in therapy for many years prior to the onset of the 3PD-related symptoms for resolution of early childhood trauma. In the last couple of years, his therapist had addressed this client's symptoms with a variety of therapeutic modalities, including CBD (Cognitive Behavioral Therapy), which has shown encouraging results in patients with 3PD (Waterson et al., 2021). Each intervention had some impact on a subset of the presenting symptoms, but the client noted that the improvements were either not significant or were not holding for a longer period of time, enough to have a lasting effect.

The Neurofeedback assessment, as it is designed for the ILF Neurofeedback approach (Othmer, 2019), included an evaluation of current symptoms and description of other symptoms he had exhibited throughout his life, and all this information was used to design a training protocol to address his specific needs. The evaluation revealed a difficult birth experience, via C-section, with possible oxygen deprivation for a brief period. He described suffering some developmental trauma, for which he had been in therapy for a long time. The client suffered several concussions, among which one occurred during a skateboarding accident when he was about 12 years old and resulted in brain injury in the occipital area. He remembers experiencing a sensation of unsteady ground for a while after riding an elevator, as well as disturbances of visual perception, such as the white board at school moving in an undulating trajectory. His pediatrician at the time checked him out and disregarded his reports. He suffered an electrocution accident at the age of 17, which left him struggling with lingering fatigue. More recently, several consecutive losses in his life brought up a high level of stress and worsened existing symptoms.

His clinical presentation before starting Neurofeedback included the following symptoms: constant sensation of stepping on unstable ground, like the sensation of riding an elevator, a continuous rocking feeling as if being in a boat, with a fluctuating intensity, and poor balance. He reported difficulty falling asleep due to perceived dizziness and constant sensation of movement as well as difficulty adjusting to changing positions from standing to sitting to lying down, which would always worsen the symptoms for a period. He also experienced intense nightmares and cluster headaches frequently, rare migraines and occasional eye pain that was very intense. Visual distortions, with reports of wavey walls closing in the road looking like an accordion after driving for a while and blurred and double vision were part of the clinical presentation as well. Tinnitus and neck pain were a constant presence; in consequence, he was very concerned about his health and afraid he would never be able to function normally again. Anxiety manifested as chest and stomach tightness, and he had a history of panic attacks related to the use of marijuana. He had a history of suicidal thoughts and more recently was struggling with intrusive, violent thoughts of wanting to harm people very close to him. Brain fog and fatigue completed the clinical presentation. The symptoms had become unbearable after the recent traumatic losses he suffered, and for a couple of years he was bedridden and completely disabled, unable to drive or even leave the house. By the time we started his Neurofeedback training, the intensity of his symptoms had decreased, mostly due to the intense work he did during vestibular rehabilitation, along with psychotherapy. He was able to drive but was still not working; any level of physical activity would result in immediate worsening of symptoms.

From a Neurofeedback standpoint, the above listed symptoms indicate this client's nervous system was presenting with deficits of arousal regulation and excesses in neuronal excitability. There are two main effects to ILF Neurofeedback training: calming and stabilizing. The calming effect impacts arousal dysregulations and related symptoms. The stabilizing effect impacts excitability and the paroxysmal symptoms resulting from deficits in inter-hemispheric coordination. Calming and stabilizing are specifically addressed by targeting certain brain areas, promoting self-regulation and resolution of symptoms (Othmer, 2019).

Trauma experienced by the developing brain results in arousal regulation deficits, pushing the brain into emergency mode, which ensures survival. In some cases, however, after a traumatic experience, the nervous system remains in fight-or-flight mode indefinitely (Van der Kolk, 2014). This interferes with optimal brain function and can manifest as physical, emotional, or mental agitation (Othmer, 2019). In this client's case, the agitation was expressed as physical anxiety (stomach and chest tightness) and neck pain, as well as emotional and mental agitation expressed as intrusive, violent thoughts, fear, and despair with respect to life prospects.

The client also presented with a variety of symptoms of instability rooted in excitability regulation deficits and related to the inability to maintain an optimal balance between excitation and inhibition at a synaptic level. Traumatic brain injuries and oxygen deprivation at birth put the brain at risk for such deficits, and in this client's case there was significant genetic vulnerability as a compounding factor (Sacks, 1992; Othmer, 2019). All these factors led to a propensity to hyper-excitability at a neuronal level, which can manifest as headaches, panic, tinnitus, and vertigo-like symptoms (Othmer, 2019).

Additionally, there were relevant traits in his family history: asthma, autoimmune disorder, thyroid disorder, anxiety, and dissociative identity disorder. Most of these findings were indicative of a genetic propensity to excitability regulation deficits.

Because of all the manifestations related to excitability dysregulation, the starting placement for this client was T3-T4, meant to provide a strong stabilizing effect and impact related symptoms. Going into session he experienced anxiety, described as nervousness and tightness in his stomach, a moderate headache, and intense rocking and swaying sensation due to the driving and walking he did to get to the appointment. Because the visual feedback influenced the swaying and rocking sensation negatively, making it more severe, we trained eyes-closed, using only auditory and tactile feedback. As the session unfolded, the optimization process revealed the more calming settings positively impacted the anxiety and the headache, which resolved by the end of the session while the rocking sensation settled down a little as well. As the optimization process continued over the next couple of sessions, the T4-P4 placement was added to provide a stronger and more precise calming effect to improve sleep and nightmares, and more specifically targeting sensory processing. The training continued over the next few sessions with T4-P4 and T3-T4, while the frequency adjustments were made to identify the optimal training frequency. Once symptoms started to improve, visual feedback was included in the training, and the rest of time we did eyes-open sessions. Changes in symptoms came quickly; he reported a remarkable positive shift in tunnel vision by session 3, although the overall rating for visual processing problems did not change significantly until later during the training Tunnel vision was something he would always experience when sensory information became overwhelming, and his brain wasn't able to process it fast enough. The improvement was directly correlated with training the parietal area on the right side, which impacts sensory processing and integration. Neck tension, headaches and nightmares or vivid dreams lessened in intensity and frequency, and during sessions he reported brain fog lifting and the teeter-totter sensation resolving, leaving him clear-minded, calmer and more stable overall by session 5. At one point he described that in between sessions, while in a new environment where dizziness and swaying started to settle in, he felt as if the brain made a choice to change that, almost as if it knew how to resolve the problems due to integrating training effects. He stated that even with known triggers his brain seemed less reactive, and recovery after worsening of symptoms was much faster since beginning neurofeedback training. He noted that the intrusive, violent thoughts had settled down significantly.

T4-Fp2 was introduced at session 10 to address emotional regulation and control and more specifically impact intrusive thoughts, anger, and the trauma related to the recent losses he had suffered. After a few sessions on this 3-placement protocol, the client reported finding his way back to an old hobby of his, playing guitar, something he hadn't done in several years. However, he also reported an increase in the frequency of headaches, so the decision was made to return to the previous protocol, and only train T4-P4 and T3-T4, with emphasis on T3-T4 for a stronger stabilizing effect. When this protocol was not enough to control the headaches, another inter-hemispheric approach to training was tried. P3-P4 was trained for sensory processing and integration problems, but T3-T4 continued to be the core of the neurofeedback protocol.

By session 18 we added yet another training area to the protocol – O1-O2 – to impact visual field deficits more specifically and the “closing in” sensation he had in very visually stimulating environments. In session he reported feeling like the brain was “working out” during this portion of the protocol. Several sessions later he realized that since the onset of severe symptoms in 2019, he tended to only see whatever was right in front of him, ignoring the rest of the visual field due to sensory overstimulation. This was now changing, and he noticed he was now taking in the full picture of the reality in front of him, which included being able to see the corners of a room, for example. The occipital placement helped settle down the dizziness and visual distortions he still used to experience in new, heavily visually stimulating environments. With sessions scheduled once per week, the client noticed the gains after training lasted longer between sessions, and he felt more stable and had more days where he felt almost symptom-free.

## Objective measurements of brain performance

In addition to tracking subjective symptoms during sessions and from session to session, a continuous performance test (CPT) (QIKtest, Bee Medic, Switzerland) was administered to provide objective information on speed, consistency of response times and accuracy before the beginning of neurofeedback training and after 20 sessions.

The QIK CPT is a computerized visual continuous performance test developed for assessing attention and impulse control. A simple visual target or non-target is presented every 2 s. During the 21-minute test, the client must press a button to respond to each target and not press for each non-target, as rapidly as possible consistent with accuracy. The results are grouped into two indices. The accuracy index consists of two scores: sustained attention and impulse control. Sustained attention combines scores resulting from omission errors and outliers (very slow responses), while impulse control combines commission errors and anticipatory responses (press of the button before being able to process and appropriately act on presented stimulus). This test is not a diagnostic tool but accurately tracks sensitive changes in brain performance that can be traced back to the intervention used during that period of time. For this client, neurofeedback was the only intervention, therefore changes in symptoms and objective measures of brain performance can be attributed to it.

**Figure 1:**
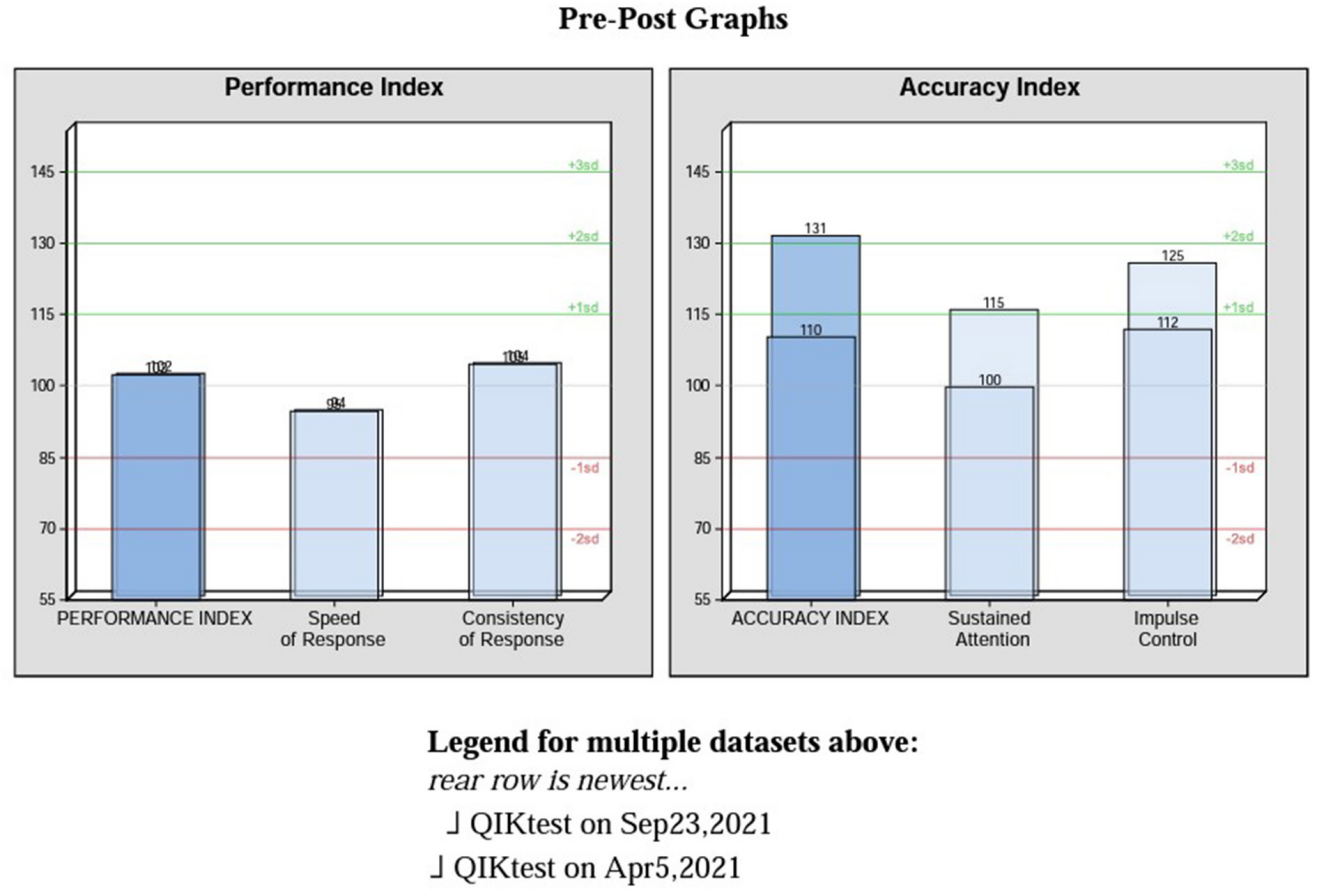
Pre-post graphs on performance and accuracy indexes.

The comparison between the data in the baseline test and the second one revealed significant improvement in accuracy, while maintaining performance scores, which supports the clinical findings of improved cognitive function and increased ability for accurate output in the absence of physical and physiological symptoms that used to disrupt his overall functioning. The accuracy index changed significantly with both sustained attention and impulse control scores falling into the above average bracket for QIK test 2 (see Figure 1).

**Figure 2:**
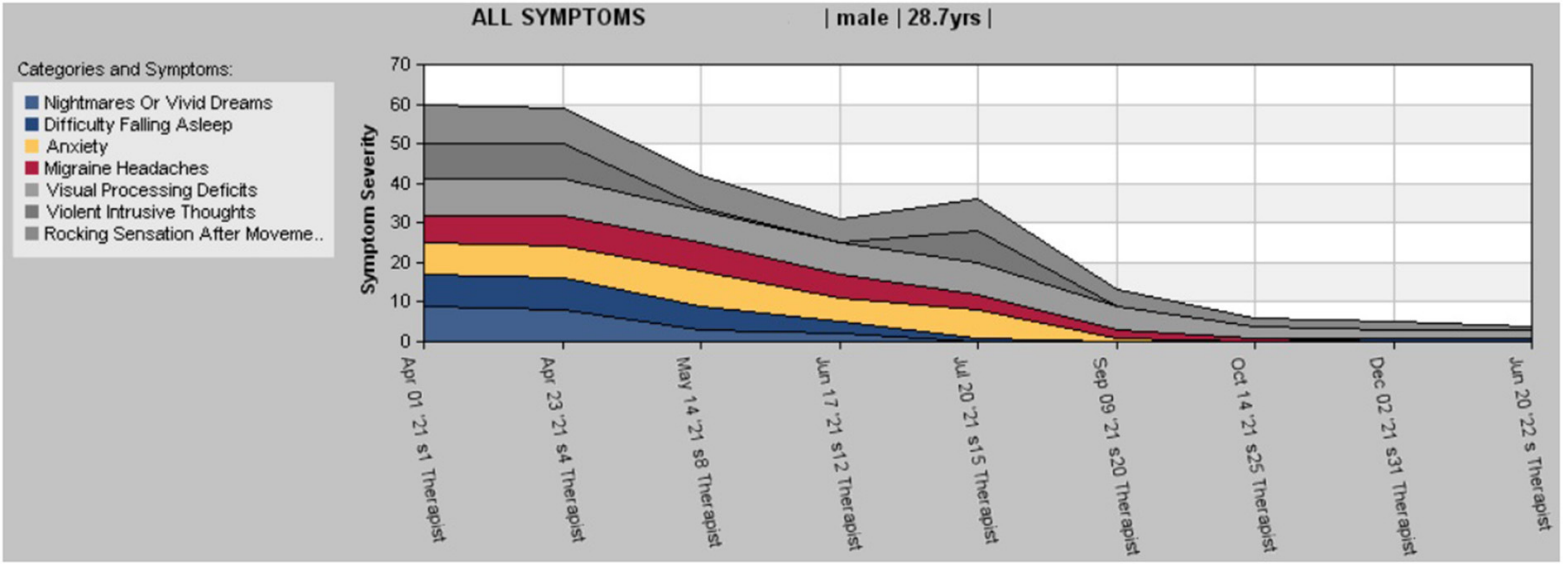
Stacked chart of symptom tracking.

While the objective measurement demonstrates improvement, symptoms were still present and needed more training. The gains continued to accrue and by session 28 new environments, even when visually and auditorily over-stimulating, were not triggering any symptoms. He could ride an elevator comfortably without anxiety or the rocking sensation coming up. By session 30 the client reported his vision being clearer, his brain feeling sharper, with no intrusive thoughts or urges and no headaches. Vertigo-like symptoms were no longer triggered by chiropractic adjustments. His sleep had settled, and no rocking or swaying were triggered by changing the position of this body. He started going out more and enjoying it, now that the symptoms were not triggered by being in unfamiliar places. With sessions spaced out 2 weeks apart, it became clear by session 32 that the gains were holding steady, and symptoms were not re-emerging, even with the usual triggers. He was now able to work more and was making plans to expand his business. During the last check-at 6 months after his last Neurofeedback session, the client stated that: “I had a positive experience with Neurofeedback and it helped a lot in other aspects of my life as well, like processing sound and vision.” The last symptom rating done at that time is comparable to the rating done at session 32, with a slight improvement in the perceived rocking sensation with movement (see Figure 2).

**Figure 3:**
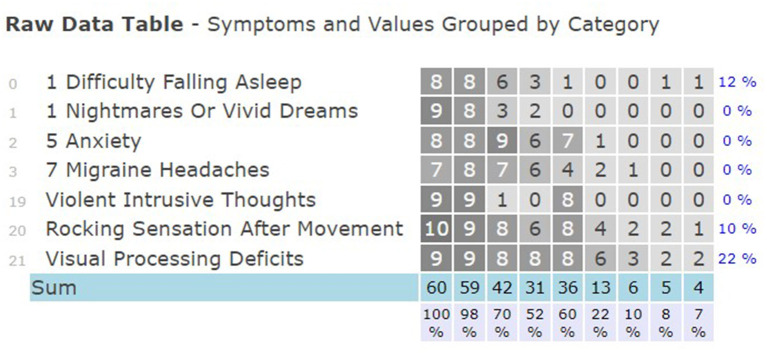
Rating of symptoms and change percentages.

The raw data table (Figure 3) displays changes in rating for each listed symptom and percentage comparing first day to last day of each symptom.

## Conclusions

Infra-low Neurofeedback is a non-invasive brain training technique, individualized to target specific neuronal circuits and improve self-regulation and allow better physiological organization of function. In the above-described case report, ILF Neurofeedback was a competent addition to this client's treatment regime, one that allowed not only symptom relief but also resolution of the core dysregulations, which in his case were related to excitability regulation and arousal regulation deficits. It is believed that this is possible due to restoration of the internal functional connectivity of the default mode and salience networks (Othmer, 2016a). The electrode placements used for this client's protocol target hubs in the default mode network, thus impacting the organization of baseline (resting) states that ensure good function (Othmer, 2016b). This allowed improved self-regulation and overall function, with significant changes in all areas of concern. While ILF Neurofeedback is a powerful intervention that can be added to a client's treatment regime to allow core regulation and increased efficacy of other interventions used in the case, outcomes will differ depending on how sensitive the client is to the approach, the clinician's understanding of symptoms and appropriate application of the method, and the communication between practitioner and client.

## Data availability statement

The original contributions presented in the study are included in the article/supplementary material, further inquiries can be directed to the corresponding authors.

## Author contributions

The author confirms being the sole contributor of this work and has approved it for publication.

## Conflict of interest

The author declare that the research was conducted in the absence of any commercial or financial relationships that could be construed as a potential conflict of interest.

## Publisher's note

All claims expressed in this article are solely those of the authors and do not necessarily represent those of their affiliated organizations, or those of the publisher, the editors and the reviewers. Any product that may be evaluated in this article, or claim that may be made by its manufacturer, is not guaranteed or endorsed by the publisher.
